# Determinants of enrollment decision in the community-based health insurance, North West Ethiopia: a case-control study

**DOI:** 10.1186/s12992-019-0535-1

**Published:** 2020-01-06

**Authors:** Getasew Taddesse, Desta Debalkie Atnafu, Asmamaw Ketemaw, Yibeltal Alemu

**Affiliations:** 1 Depatment of Health System and Health Economics, School of Public Health, College of Medicine and Health Sciences, Bahir Dar University, Bahir Dar, Ethiopia; 2 Depatment of Health Informatics, School of Public Health, College of Medicine and Health Sciences, Bahir Dar University, Bahir Dar, Ethiopia; 3 Department of Reproductive Health and Population Studies, School of Public Health, College of Medicine and Health Sciences, Bahir Dar University, Bahir Dar, Ethiopia

**Keywords:** Health, Insurance, Enrollment, Determinant, Ethiopia

## Abstract

**Objective:**

To identify the determinants for enrollment decision in the community-based health insurance program among informal economic sector-engaged societies, North West Ethiopia.

**Method:**

Unmatched case-control study was conducted on 148 cases (member-to-insurance) and 148 controls (not-member-to-insurance program) from September 1 to October 30,2016. To select the villages and households, stratified then simple random sampling method was employed respectively. The data were entered in to Epi-info version 7 and exported to SPSS version 20 for analysis. Descriptive statistics, bi-variable, and multi-variable logistic regression analyses were computed to describe the study objectives and identify the determinants of enrolment decision for the insurance program. Odds ratio at 95% CI was used to describe the association between the independent and outcome variables.

**Results:**

A total of 296 respondents (148 cases and 148 controls) were employed. The mean age for both cases and controls were 42 ± 11.73 and 40 ± 11.37 years respectively. Majority of respondents were males (87.2% for cases and 79% for controls). Family size between 4 and 6 (AOR = 2.26; 95% CI: 1.04, 4.89), history of illness by household (AOR = 3.24; 95% CI: 1.68, 6.24), perceived amount of membership contribution was medium (AOR = 2.3; 95% CI: 1.23, 4.26), being married (AOR = 6; 95% CI:1.43, 10.18) and trust on program (AOR = 4.79; 95% CI: 2.40, 9.55) were independent determinants for increased enrollment decision in the community-based health insurance. While, being merchant (AOR = 0.07; 95% CI: 0.09, 0.6) decreased the enrollment decision.

**Conclusion:**

Societies’ enrollment decision to community-based health insurance program was determined by demographic, social, economic and political factors. Households with large family sizes and farmers in the informal sector should be given maximal attention for intensifying enrollment decision in the insurance program.

## Background

Health security is increasingly being recognized as an integral part of poverty reduction effort [1]. Many lower and middle-income countries (LMICs) have not been able to fulfill equitable healthcare needs of their citizens. They faced challenges in raising sufficient funds to finance health services [2, 3]. Consequently; these days, they have been promoting community-based health insurance program (CBHI) as a means of financing healthcare [3]. However, it had not been brought significant impact on the accessibility of healthcare because of the low level of members’ enrollment rate [3, 4].

Developing countries account for 84% of the global population from which about 50% and more live under poverty. About 1.3 billion are rural poor informal sector workers that produced 20% of the domestic product. Additionally, 90% of global disease burden was born by such countries [4–6]. Although, countries had agreed in world health organization (WHO) general assembly of 2005 to achieve universal health coverage (UHC) through developing risk-pooling mechanism (health insurance program) by reducing out-of-pocket payments (OPP), the experience of spending money for healthcare consumption still existed low, less than 12% [4–7].

As a result, annually around 808 million peoples faced catastrophic direct health expenditure at a percentage of 10 income health expenditure ratio [7]. Among peoples with catastrophic direct healthcare expenditures, 100 million were impoverished and over 90% of them had occurred in LMICs [7, 8]. The amount of out-of-pocket spending was increased through time in Ethiopia and still one of the barriers to accessing healthcare in the country [9]. In the Ethiopia, 37% of finance for health was generated from households’ direct payments at time of healthcare utilization [9–11]. The government health spending in Ethiopia was limited to below 5% of the total gross domestic product (GDP), much lower than threshold of Alma Ata declaration (15%) that every nation agreed to allocate [12]. According to the direction shown in Ethiopian health policy endorsed in 1993, ministry of health of the country developed healthcare financing strategy with a great emphasis on health insurance agenda [12, 13]. In the health sector transformation plan II, Ethiopian government also targeted a reduction of out-of-pocket expenditure from 37% to less than 15%, and catastrophic out-of- pocket health expenditure from 3 to 2.5% by designing and implementing CBHI program on 80% of districts. In the strategic plan, about 80% of the communities were included to have enrollment decision for membership in the program [10]. However, with the existing regional variation; only 5% of the communities were enrolled to the program, as reported in the Ethiopian demographic and health survey (EDHS), 2016 [14]. The same was also reported by the performance statistics of Africa that from 900 million eligible people to be a member for community insurance, only two million or 0.2% were actually enrolled to the program [2, 5, 15]. This assured that the amount (status) of financial pool for healthcare was minimal because of the fact that lower-income countries were faced with challenges to sustain the CBHI scheme among eligible populations and the scheme served only small proportion of them [16, 17].

According to 2016 report of Ethiopian health insurance agency (EHIA), CBHI scheme has been started piloting in 2011and only 30% of the communities decided to enroll in CBHI program at the time of launching the pilot [12]. On the other hand, the scheme was characterized by challenges not only low membership ratio but also fluctuation of membership decision which led members like drop out from the program once they had been already joined [1–3, 18]. This was confirmed by facts found in the study area, that the scheme enrolment rate was registered to be 33% while the drop outs (refusal) was 31% [19]. The study was conducted by referring program policy documents and strategies like program awareness creation process and means, administration process from village up to district level, pre-defined benefit packages and health service utilization process, trusts on policy as well as on the development of the programs and its sustainability [20]. The study would act as a baseline data source for policy agenda setting and identified the potential barriors for enrollment decision where the enrollment rate was low and on the other hand the drop outs was higher. There for, this study aims to figure out why all the targeted eligible households did not participate and enroll into the existing scheme?

## Method

### Study setting and period

The study was conducted in South Achefer district between September to October 2016. South Achefere is 500 km North East of the capital Addis Ababa. The district was made up of 20 Villages (18 rural and 2 urban villages). Based on the 2007 national census conducted by the Central Statistical Agency of Ethiopia (CSA), the projected total households of the district for 2016 were estimated to be 36,204. About 12,612 (35%) households were enrolled to CBHI. Health service coverage of the district was reached to 100% since 2015. There are 8 health centers, 20 health posts and one primary hospital that give health service for the dwellers. The majority of the inhabitants are subsistent farmers (85%), whereas the remaining includes merchants (12%) and others (3%) [19].

### Study design

An unmatched case-control study.

### Sample size estimation

The sample size was computed using Epi Info™ 7 software on the basis of the following assumptions: 80% statistical power with a level of significance at 5%, Odds ratio of 2 and a case to control ratio of 1:1 [16]. The estimated final sample size was 148 for both cases and controls using the double-proportions formula. The sample size was calculated for variables: history of illness, occupation, premium loading. Hence, the largest sample size among the exposure variables was taken.

### Sampling methods and procedure

The study was conducted using stratified random sampling method. The study population was categorized into four strata based on the existing administration heterogeneity. Each stratum had five villages in it. However, we considered only 10 villages for this study by using lottery method. The sample size computed was allocated to each villages based on probability proportional to size, where numbers of households were used as a measure of size. The required numbers of households in each of the selected villages were drawn by simple random sampling methods from the frame found in CBHI registrar for cases and health extension family folder for controls maintained by health extension workers.

### Data collection tools and techniques

A structured questionnaire was used to collect data via face-to-face interview from the head of selected households. The questionnaire was prepared first in English and translated to Amharic and then again translated back to English to check its consistency. Five data collectors, who were in charge of minimum diploma certification, were recruited for data collection. To maintain the quality of data, pre-test was conducted on 5% of the sample size in other villages which were not included in the study. Training was also provided for both data collectors and supervisors for one day basis.

### Operational definitions

#### CBHI membership:

When households are joining in to CBHI by paying the pre-set amount of membership contribution (money) and becoming eligible to utilize health services.

#### Perceived affordability of membership contributions:

Perceptions of households on the pre-set contribution rate of the program fixed by the scheme ($6.72 per annum).

#### Perceived quality of services:

The extent of the community’s views on the quality of health service delivery and is measured by one item, two-point Likert scale questions.

#### Trust on CBHI program

Can be defined through variables such as: composition of scheme executive in team, deliverability of benefit package and capacity of contract health providers.

### Data analysis

Data were entered into Epi-info V.7 and analysis was performed with SPSS V.20. Descriptive statistics was computed to describe the study objectives in terms of appropriate variables. Binary and multi-variable logistic regression analyses were performed to identify the most important variables which could determine enrollment decision in CBHI scheme. Variables with a *p*-value of ≤0.2 on binary logistic regression analysis were entered and further computed on the multi-variable logistic regression model. Associations between the study and outcome variables were described using odds ratio at 95% CI. The Hosmer–Lemeshow test was checked and the model adequately fit to the data at the *p*-value > 0.05.

### Ethical considerations

The data collection was carried out after ethical clearance and letter of permission have been obtained from ethical board of Addis Continental Institute of Public Health (ACIPH) and district administration for CBHI scheme respectively. Verbal consents were also taken by data collectors from all respondents.

## Results

### Socio – demographic and enrollment profiles of respondents

A total of 296 (148 non-members & 148 members) respondents consented to participate in this study, resulting in an overall response rate of 100% (both for cases and controls). The mean ages of both cases and controls were 42 (±11.73 SD) and 40 (±11.73 SD) years respectively. Among the total households interviewed, majority (83%) were headed by males.

Family size, occupation, marital status, income, participation in social practice, history of illness and perceived quality of services showed significant difference between the members versus the non members households, but the variables age, sex, educational status etc. did not show variation between the case and control households (Table 1).

**Table 1:** Socio-demographic and CBHI enrollment characteristics (profiles) of study participants in North Western Ethiopia, October 2016

Characteristics	Not Member to CBHI (Controls) (%)	Member to CBHI (Cases) (%)	Chi square, *p*-value
Sex			
Male	117 (39.5)	129 (43.6)	x^2^ = 3.465, *p*-value = 0.063
Female	31 (10.5)	19 (6.4)	
Age of heads			
< 35	43 (14.5)	32 (10.8)	x^2^ = 2.43, *p*-value = 0.29
35–51	81 (27.4)	86 (29.1)	
> 51	24 (8.1)	30 (10.1)	
Educational status			
Unable to read and write	44 (14.9)	40 (13.5)	x^2^ = 4.24, *p*-value = 0.374
Have informal education	55 (18.6)	55 (18.6)	
Primary & junior education [1–8]	27 (9.1)	39 (13.2)	
Secondary education [9–12]	13 (4.4)	9 (3.0)	
Above 12^ th^ grade	9 (3.0)	5 (1.7)	
Occupation			
Farmer	122 (41.2)	134 (45.3)	x^2^ = 11.04, *p*-value = 0.004**
Merchant	22 (7.4)	6 (2.0)	
Others	4 (1.4)	8 (2.7)	
Family size			
< 3	37 (12.5)	34 (11.5)	x^2^ = 17.86, *p*-value = 0.001**
[3–4]	15 (5.1)	14 (4.7)	
[4–6]	47 (15.9)	69 (23.3)	
> 6	19 (6.4)	31 (10.5)	
Marital status			
Single	29 (9.8)	16 (5.4)	x^2^ = 5.69, *p*-value = 0.05*
Married	116 (39.2)	125 (42.2)	
Divorced & widowed	3 (1.0)	7 (2.4)	
Household annual income			
< 8000	50 (16.9)	27 (9.1)	x^2^ = 9.37, *p*-value = 0.02*
8001–16,000	38 (12.8)	48 (16.2)	
16,001–28,000	29 (9.8)	37 (12.5)	
> 28,000	31 (10.5)	36 (12.2)	
Participate in social practice			
No	19 (6.4)	3 (1.0)	x^2^ = 12.57, *p*-value = 0.001**
Yes	126 (42.6)	145 (49.0)	
Previous (history) of illness			
No	59 (19.9)	34 (11.48)	x^2^ = 9.799, *p*-value = 0.002**
Yes	89 (30.06)	114 (38.5)	
Awareness of the program			
Not aware	110 (37.16)	101 (34.12)	x^2^ = 1.34, *p*-value = 0.25
Aware	38 (12.8)	47 (15.88)	
Perceived quality of service			
Not quality	85 (28.72)	70 (23.65)	x^2^ = 3.05, *p*-value = 0.05*
Quality	63 (21.28)	78 (26.35)	
Expected future health status			
Good	115 (38.85)	120 (40.54)	x^2^ = 1.05, *p*-value = 0.60
Worse	7 (2.36)	4 (1.35)	
I can’t expect	26 (8.78)	24 (8.11)	

Significant at * = 0.05; ** = 0.01; *** = 0.001

### Bi-variable analysis

Merchant study subjects (COR = 0.14; 95% CI: 0.03,0.61) were less likely to enroll in to CBHI than subjects who were farmers. Those engage in social practices (COR = 7.11; 95% CI: 2.05, 24.61), richer to pay fees (COR=2.5; 95%CI:1.1,4.21), married (COR=1.95; 95%CI:1.01,3.78) and had large family size (>6) (COR=3.22;95%CI:1.39,6.3), were more likely to decide and join CBHI program. However, age, educational status and sex were not correlated with enrolment decision in CBHI scheme (Table 2).

**Table 2:** Socio-demographic determinants for CBHI enrolment decision in North Western Ethiopia, October 2016

Characteristics	Non-Member to CBHI (Controls) (%)	Member to CBHI (Cases) (%)	Crude OR (95% CI)
Sex			
Male	117 (39.5)	129 (43.6)	0.55 (0.29–1.04)
Female	31 (10.5)	19 (6.4)	1.00
Age of heads			
< 35	43 (14.5)	32 (10.8)	1.00
[35–51]	81 (27.4)	86 (29.1)	1.43(0.82–2.47)
> 51	24 (8.1)	30 (10.1)	1.68(0.83 - 3.40)
Educational status			
Unable to read and write	44 (14.9)	40 (13.5)	1.00
Have informal education	55 (18.6)	55 (18.6)	1.20(0.62–1.94)
Primary & junior education [1–8]	27 (9.1)	39 (13.2)	1.59(0.82–3.05)
Secondary education [9–12]	13 (4.4)	9 (3.0)	0.76(0.29–1.97)
Above 12	9 (3.0)	5 (1.7)	0.61(0.19–1.98)
Occupation			
Farmer	122 (41.2)	134 (45.3)	0.55(0.16–1.87)
Merchant	22 (7.4)	6 (2.0)	0.14(0.03–0.61)*
Others	4 (1.4)	8 (2.7)	1.00
Family size			
< 3	37 (12.5)	34 (11.5)	1.00
3–4	15 (5.1)	14 (4.7)	1.84(0.79–4.25)
4–6	47 (15.9)	69 (23.3)	2.89(1.66–5.04)*
> 6	19 (6.4)	31 (10.5)	3.22(1.59–6.50)*
Marital status			
Single	29 (9.8)	16 (5.4)	1.00
Married	116 (39.2)	125 (42.2)	1.95(1.01–3.78)*
Divorced& Widowed	3 (1.0)	7 (2.4)	4.23(0.96–18.65)
Household annual income			
< 8000	50 (16.9)	27 (9.1)	1.00
8001–16,000	38 (12.8)	48 (16.2)	2.34(1.24–4.40)*
16,001–28,000	29 (9.8)	37 (12.5)	2.36(1.20–4.64)*
> 28,000	31 (10.5)	36 (12.2)	2.15(1.10–4.21)*
Participate in social practice			
No	19 (6.4)	3 (1.0)	1.00
Yes	126 (42.6)	145 (49.0)	7.11(2.05–24.61)*

*significant at p-value 0.05

Even though, in bi-variable logistic regression analysis, participants who perceived the services delivered good quality were 1.5 times more likely to have a decision on CBHI enrollment than subjects whose perception was poor quality, the association was not statistically significant. Perceived affordability of membership contribution (COR = 2.95; 95% CI: 1.77, 4.95) and trust on the scheme (COR = 3.81; 95% CI: 2.25, 6.46) had an increasing effect on enrollment decision for CBHI program. Study participants who had history of illness in their households (COR = 2.22; 95% CI: 1.34, 3.68) were more likely to enroll in to CBHI program. However, time when membership payments made was not a determinant for enrollment decision (Table 3).

**Table 3:** CBHI-program related determinants for CBHI enrollment decision in North Western Ethiopia, October 2016

Characteristics	Non-Member to CBHI (Controls) (%)	Member to CBHI (Cases) (%)	Crude OR (95 CI)
Time when membership payments made			
Not-appropriate	45(57.69)	33 (42.31)	1.00
Appropriate	103 (47.03)	116 (52.97)	0.65(0.39–1.10)
Perceived quality of service			
Not quality	85 (54.84)	70 (44.16)	1.00
Quality	63(44.68)	78(55.32)	1.50(0.95–2.38)
Perceived affordability of membership contribution			
Expensive	70(65.42)	37(34.58)	1.00
Medium	61 (39.1)	95 (60.9)	2.95(1.77–4.92)*
Low	17(51.5)	16(48.5)	1.78(0.80–3.93)
Trust in the scheme			
No	68(71.58)	28 (28.42)	1.00
Yes	80 (39.8)	121(60.2)	3.81(2.25–6.46)*
History of illness			
No	59 (63.4)	34 (36.6)	1.00
Yes	89 (43.8)	114(56.2)	2.22(1.34–3.68)

*significant at p-value 0.05

### Multivariable analysis

Being merchant (AOR = 0.07; 95% CI: 0.09, 0.60) was an independent predictor for decreased decision to enroll in CBHI scheme. Being married (AOR = 6.0; 95% CI: 1.43, 10.18), family size between 4 and 6 (AOR = 2.26; 95% CI: 1.04, 4.89), history of illness by households (AOR = 3.24; 95% CI: 1.68, 6.24), perceived affordability of membership contribution was medium (AOR = 2.3; 95% CI: 1.23, 4.26) and trust on the program (AOR = 4.79; 95% CI: 2.40, 9.55) were independent determinants for increased enrollment decision in the CBHI program. However, participation in preexisting social practice and annual house hold income was not found significant in the final multivariable analysis (Table 4).

**Table 4:** Independent determinants for CBHI enrollment decisions among households in Northwest, Ethiopia, October 2016

Characteristics	COR (95% CI)	AOR (95% CI)
Occupation		
Farmer	0.55(0.16–1.87)	0.58(0.08–3.95)
Merchant	0.14(0.03–0.61)*	0.07(0.09–0.60) *
Others	1.00	1.00
History of illness		
No	1.00	1.00
Yes	2.22(1.34–3.68)*	3.24(1.68–6.24)*
Trust in the scheme		
No	1.00	1.00
Yes	3.81(2.25–6.46)*	4.79 (2.4–9.55) *
Perceived affordability of membership contribution		
Expensive	1.00	1.00
Medium	2.95(1.77–4.92*)	2.30(1.23–4.26) *
Low	1.78(0.80–3.93)	1.42(0.54–3.75)
Family size		
< 3	1.00	1.00
[3–4]	1.84(0.79–4.25)	1.56(0.55–4.38)
[4–6]	2.89(1.66–5.04)*	2.26(1.04–4.89) *
> 6	3.22(1.59–6.50)*	2.21(0.86–5.71)
Marital status		
Single	1.00	1.00
Married	1.95(1.01–3.78)*	6(1.43–10.18) *
Divorced & Widowed	4.23(0.96–18.65)	0.77(0.28–2.14)

*significant at 0.05

## Discussion

Limited availability of health resources and its inequitablity as well as inefficient allocation, and the existing direct payment system of it at the time of services are the main obstacles to achieve UHC, as identified by WHO [12]. The total health spending in Ethiopia has been increasing since 2004. However, the expenditures for maintenance of health in the country is still considered under-financed and said to be low when compared to other Sub-Saharan countries [12]. Hence; in 1998, Ethiopia has endorsed healthcare financing strategy as a means of collecting additional resources for health in order to ensure sustainable, equitable and quality health service delivery [13]. The government of Ethiopia, in its second health sector transformation plan (HSTP II), has put an insight: reduction of out-of-pocket (OPP) expenditures to 15% from the total household expenditure, catastrophic health expenditure from 3 to 2.5%, increasing per capital health service utilization rate from 0.48 to 2, incorporating 80% districts and 80% of the communities in CBHI program targeting the year 2020 [10]. Nevertheless, until the time of this study under taken, only 4.5% were enrolled to the CBHI program [21].

The study revealed that size of the family in household was a determinant for CBHI membership decision. This finding of the study is mirrored with a study conducted in India [3] and Nigeria [2]. This might be happened due to economics rationality of human beings in that peoples are always risk-aversers in most of the cases. The probability of getting health problem in a family depends on its size found in it. Consequently, the more the household have larger family size, the likelihood of being ill at least one member in it would be higher and the more the tendency to join into risk pooling institutions (associations). On the other hand, the result found in the current study contradicted the study which conducted in Burkina Faso [16, 22, 23]. This difference could be attributed to mechanisms of calculating expected amount of contribution paid from every household for membership in an insurance package. In Burkina Faso, the membership contribution in an insurance scheme was estimated per head in household, which levied large amount of money as family size increased, contrary to what has happened in Ethiopia [20]. This might create financial burden on the families with large size and that is why the enrollment decision in such kind of families was eroded. In Ethiopia, membership contribution was computed per household irrespective of family size (flat-rate payment system). This connection was also highly strengthened by the results found from the current study and existing literatures [24, 25] which revealed being married increased households’ enrollment decision to any insurance institution. This could be explained by the fact that the payment policy of the households’ membership contribution in Ethiopia has not taken into consideration the marital status [20]. The premium loading, which has been rated, was against the household itself rather than the number of people found in it.

Households’ previous history of health problem was a likely determinant for enrollment decision, consistent with previous studies [1, 2]. The studies conducted in Ghana and India had shown that families with an illness at least in one member in the past one year were more likely be willing to enroll into insurance scheme than their counterparts and this confirmed the effect of adverse selection on enrollment decision. The more households experienced health problems, the more expenses they would incur for each healthcare consumed and households decide to enroll into any risk-poling programs so as to transfer their risk to an insurance company. This is also because of fearing future health costs when family members might become sick [1, 2]. However, this finding is contradictory to the study conducted in Burkina Faso [26–28]. The inconsistency might be due to the fact that households’ apprehensions (fears) about the compromise of quality of service they would be consuming during the enrollment time. The difference was also explained in that the CBHI program holders in Burkina Faso could make contractual agreement with less-trusted healthcare facilities. In other words, for the same level of healthcare delivery, there might not have been significant difference in the amount they will contribute to the scheme membership and the amount they would pay to health facility at a time of sickness. In the area where this study was undertaken, the premium loading was highly-subsidized by the government while private consumption of healthcare service was expensive is also the likely explanation for this variance.

In any activity, trusting the desirable end result of a proposed decision such as launching insurance strategy is a prerequisite to accomplish anything chosen about. The personnel involved in the scheme’s administration and the decision process as well as the way the government leads/ handles the program should be trusted by the community. This assertion was actually proven by the findings of the current study such that trusting the CBHI scheme was a determinant for increased enrollment decision in the insurance program. Other studies conducted in Cameron, Nigeria, Cambodia and Ghana also reported the issue in the same way what has found in the current study [2, 5, 29].

Type of occupation has a significant impact on enrollment decision of any insurance compendium. In other words a person, who engaged in work and generated higher amount of income, had a higher probability of being membership to the insurance scheme [16]. Similarly, some jobs are also occupationally unfit and probability of illness to an employee increased and because of adverse selection employee by the time wanted to be on the safe zone by joining into insurance. The result of the study showed that occupational difference had an impact on communities’ decision for enrolment, similar to previous studies [3, 16, 29]. In study area, the merchants were concentrated in center of villages and around the town. This made the merchants ease of accessing alternative quality healthcare services other than facilities contractual by the insurance company and thus prefer better health institution during time of healthcare use. In the town, there are a number of quality private owned health service providers where each of them did not have any agreement with the CBHI program coordinators. The CBHI scheme facilitators took agreement with only public health facilities in which unlikely preferable by most of the merchants because of patient loads and substandard care. For this reason, most merchants were doubtful to join the program so as to widen up their alternatives for getting better care and management of illness during time of healthcare need.

In Ethiopian CBHI scheme, the contribution (premium) was collected from the households at the pre-set flat-rate amount. That is equal amount of money was levied to everyone without taking into consideration any characteristics of the households including affluence or poverty and family size etc. However, when the contribution rate was made flat-rate automatically; it became more regressive irrespective of households’ income status. Thus, it was expensive for the poor and laid financial burden on them. In similar kind of insurance packages as well, such kind of characteristics of the scheme will restrict the rate of enrollment decision especially in the case of the poor. Our study also highly supported this reality in that households with medium perceived affordability of contribution were more likely to enroll into insurance scheme as compared to its counter parts. Similarly, results of the studies conducted in Ghana [1], Mali [16] and Senegal [16] reported that flat-rate system premium loading circumscribed enrolment decision of the poor [1, 18]. This study was not conducted without any limitations although we tried to minimize it. Information and recall bias related household income and perception might affect accuracy of the study.

## Conclusion

Society’s enrollment decision in community-based health insurance program was determined by demographic, social, economic and political factors. Households with large family size and farmers in the informal sector should be given maximal attention for increasing enrollment decision in the insurance program. Thus, the CBHI program coordinators should rate the amount of membership contribution based on family size to protect the scheme from bankruptcy and to attract, engage and widen the members (out of agriculture sector) by making the service provision more confortable. This study also reaffirmed that standardizing affordability of amount of membership contribution by households and building their trust on the program are some of the best strategies for increasing enrollment decision in informal sectors. Community mobilization and awareness creation of CBHI was also commendable. The findings of the study also provide impetus to intensify the influence of adverse selection on such scheme enrollment rate. The insurance policy makers should avoid immediate claims (introduce waiting time for enrollee to get service after being registered). As a result, much emphasis has to be given to households with an experience of previous illness during the time of initiation, expansion and promotion of the program.

## Data Availability

Data of this study are available without restriction. Contact to this e-mail: destad2a@gmail.com when needed.

## Abbreviations

ACIPH: Addis Continental Institute of Public Health
AOR: Adjusted Odds ratio
AOR: Adjusted odds ratio
CBHI: Community based health insurance
CI: Confidence interval
COR: Crude odd ratio
CSA: Central statistical authority
EDHS: Ethiopian demographic and health survey
EHIA: Ethiopian health insurance agency
GDP: Gross domestic product
HSTP: Health sector transformation plan
LMICs: Lower and middle income countries
OPP: Out of pocket payment
SD: Standard
SPSS: Statistical package for Social Science
UHC: Universal health coverage
WHO: World health organization
× ^2^: Chi-squire test
